# Regeneration in Aesthetic Medicine: Mechanisms, Evidence, and Clinical Boundaries

**DOI:** 10.1111/jocd.70669

**Published:** 2026-01-23

**Authors:** Antony de Paula Barbosa

**Affiliations:** ^1^ Department of Research & Development Antony Barbosa Institute Belo Horizonte Brazil; ^2^ Faculty of Pharmacy Pontifícia Universidade Católica de Minas Gerais (PUC‐Minas) Belo Horizonte Brazil

**Keywords:** collagen biostimulators, dermal–hypodermal unit, extracellular matrix remodeling, mechanotransduction, regenerative aesthetics, skin regeneration, subcutaneous adipose tissue

## Abstract

**Background:**

Regeneration has emerged as a key concept in aesthetic medicine as the field evolves from predominantly volumetric correction toward biologically oriented strategies aimed at improving tissue quality, function, and long‐term structural integrity. However, the widespread use of the term “regenerative” has often been used without biological precision, leading to conceptual overlap with repair, remodeling, and biostimulation. A critical evaluation of the biological basis and clinical evidence supporting regenerative claims is needed.

**Objective:**

To critically synthesize current biological, translational, and clinical evidence related to skin regeneration in aesthetic medicine, with emphasis on extracellular matrix remodeling, immune modulation, mechanotransduction, and dermal–hypodermal integration, and to contextualize the regenerative potential and limitations of commonly used biomaterials.

**Methods:**

A narrative review was conducted based on experimental studies, translational research, narrative and systematic reviews, and clinical investigations cited in the reference set. Evidence was qualitatively analyzed focusing on mechanisms of action, tissue‐level interactions, immune responses, extracellular matrix dynamics, involvement of subcutaneous adipose compartments, and durability of clinical outcomes.

**Results:**

Cutaneous regeneration is a multilevel functional process driven by coordinated extracellular matrix reorganization, controlled inflammation, mechanotransduction, angiogenesis, and dermal–hypodermal crosstalk. Particulate collagen biostimulators (poly‐l‐lactic acid, poly‐d,l‐lactic acid, calcium hydroxyapatite, and polycaprolactone) demonstrate the most consistent evidence for sustained functional remodeling, with poly‐l‐lactic acid showing the strongest longitudinal and histological support. Hyaluronic acid–based fillers and skinboosters primarily act as microenvironmental modulators with limited regenerative depth. Polydioxanone threads induce localized mechanobiological remodeling that is highly technique dependent. Biological bioregenerators, including polynucleotides, polydeoxyribonucleotides, and extracellular vesicles, show strong mechanistic plausibility but limited and heterogeneous clinical evidence.

**Conclusion:**

Regeneration in aesthetic medicine should be defined by biological mechanism, functional integration, and durability rather than by transient morphological change. A biologically accountable and evidence‐based framework is essential for responsible clinical application.

## 1 Introduction

The skin is a highly specialized organ whose biological relevance extends far beyond its classical definition as a physical barrier. It represents a dynamic interface between the external environment and the internal physiological milieu, integrating structural, immune, metabolic, and endocrine functions essential for tissue homeostasis and adaptation. This complexity is reflected in its organization into three intimately interconnected compartments—epidermis, dermis, and hypodermis—which communicate continuously through biochemical mediators, mechanical forces, and immunological signaling pathways. As a consequence, cutaneous regeneration cannot be attributed to the activity of a single cell type or tissue layer, but rather emerges from coordinated interactions among keratinocytes, fibroblasts, endothelial cells, adipocytes, and immune cells [1, 2, 3].

Within this integrated system, the dermal extracellular matrix (ECM) plays a central biological role. Beyond providing mechanical support, the ECM functions as an active and dynamic signaling platform that regulates cell adhesion, migration, proliferation, and differentiation. By storing and releasing growth factors, shaping cytokine gradients, and transducing mechanical stimuli into biochemical signals, the ECM governs both physiological tissue renewal and responses to injury [4, 5, 6]. Accordingly, ECM remodeling is now recognized as a primary driver of morphogenesis, repair, and regeneration, rather than a passive or secondary phenomenon [7].

Aging profoundly alters this finely regulated microenvironment. Both intrinsic aging and cumulative exposure to extrinsic stressors—particularly ultraviolet radiation—lead to progressive fragmentation of collagen fibers, disorganization of elastic networks, increased matrix metalloproteinase activity, and a decline in fibroblast biosynthetic capacity. Notably, these changes reflect not merely a reduction in collagen content, but a qualitative disruption of matrix architecture and cell–matrix communication, resulting in impaired mechanotransduction and reduced regenerative responsiveness [8, 9]. As a result, the aged dermis becomes mechanically fragile and biologically less adaptive, favoring inefficient repair or fibrotic remodeling rather than restoration of native tissue structure and function [10].

Biologically, regeneration must be clearly distinguished from classical scar‐based repair. While repair restores tissue continuity, it often does so through disorganized collagen deposition and incomplete reconstruction of native architecture. Regeneration, in contrast, is defined by a temporally and spatially orchestrated sequence of events that includes controlled inflammation, ECM remodeling, angiogenesis, fibroblast activation, and in selected contexts, recruitment of resident progenitor populations [10, 11]. This distinction is particularly relevant in aesthetic and reconstructive medicine, where durable clinical improvement increasingly correlates with restoration of tissue quality, elasticity, and biomechanical behavior rather than with transient volumetric correction alone [8].

A critical conceptual advance in recent years has been the recognition that inflammation is not inherently antagonistic to regeneration. On the contrary, a controlled and self‐limited inflammatory response is a prerequisite for effective tissue remodeling. Biomaterial‐induced inflammation, when subclinical and properly regulated, initiates innate immune cascades that recruit fibroblasts, stimulate angiogenesis and promote ECM reorganization [12, 13]. In this context, macrophage polarization has emerged as a pivotal determinant of regenerative outcomes, with a transition toward pro‐regenerative (M2‐like) phenotypes supporting organized collagen deposition, vascular maturation and resolution of inflammation [14, 15].

Alongside advances in immunobiology, the hypodermis is increasingly recognized as a key regulator of skin regeneration. Long regarded primarily as a volumetric and metabolic reservoir, adipose tissue is now understood to function as an endocrine and paracrine organ that actively influences dermal homeostasis. Dermal and subcutaneous white adipose tissue contribute to regenerative niches through adipokines, growth factors, and resident progenitor cells, modulating fibroblast activity, angiogenesis, and immune responses [16, 17]. This dermal–hypodermal axis provides a biological explanation for the sustained improvements in skin quality observed with regenerative interventions that extend beyond the dermis alone.

These biological insights have reshaped the conceptual framework of aesthetic medicine. Instead of treating aging as a purely geometric problem to be corrected by volume replacement or tissue repositioning, contemporary approaches increasingly align with principles of translational regenerative medicine, seeking to restore tissue function and microenvironmental balance by activating endogenous repair pathways [8, 16]. This shift underpins the concept of regenerative aesthetics, which prioritizes biological sustainability, tissue quality, and functional integration over isolated corrective outcomes.

Within this framework, collagen biostimulators occupy a central position. These agents are defined as biomaterials capable of inducing neocollagenesis and dermal or subcutaneous remodeling through a controlled, subclinical inflammatory response triggered by their interaction with host tissue [18]. Following implantation, they promote recruitment of innate immune cells, activation of fibroblasts and progressive deposition of collagen—predominantly types I and III—leading to reinforcement of the ECM and improvement of tissue biomechanics [12, 19]. Increasingly, their clinical efficacy is understood to depend not only on the amount of collagen produced, but on its organization, maturation, and functional integration within the existing matrix [20, 21].

Concurrently, a complementary category of agents referred to as bioregenerators has gained prominence. Unlike particulate biostimulators that rely primarily on scaffold‐induced remodeling, bioregenerators act predominantly through bioactive signaling mechanisms, modulating inflammation, oxidative stress, angiogenesis, and cellular metabolism. Polynucleotides, polydeoxyribonucleotide, and extracellular vesicles exemplify this approach, promoting tissue regeneration through purinergic signaling, intercellular communication, and microenvironmental modulation rather than through mechanical support alone [22, 23, 24].

Taken together, these developments support a unified view of skin regeneration as a biological continuum. Collagen biostimulators and bioregenerators should not be viewed as competing modalities, but as complementary tools acting at different levels of the regenerative cascade. Their rational integration—guided by tissue biology, immune status and dermal–hypodermal interactions—supports a biologically coherent approach to contemporary regenerative aesthetic and reconstructive practice.

In this review, the term *regeneration* is used to describe a biologically driven process involving coordinated extracellular matrix remodeling, immune regulation, mechanotransduction, and functional tissue integration. The adjective *regenerative* is applied exclusively to biological processes, pathways, or responses and not as a categorical label for aesthetic products or devices. This distinction is essential to avoid conceptual overlap between regeneration, repair, remodeling, and biostimulation and to ensure biological and clinical precision in the context of aesthetic medicine.

## 2 Methodology

### 2.1 Study Design

This manuscript was developed as a narrative review with the objective of critically synthesizing and clinically contextualizing the current biological and translational understanding of skin regeneration, collagen biostimulators and bioregenerative agents within aesthetic and reconstructive medicine. A narrative design was chosen to allow integrative interpretation of heterogeneous evidence derived from experimental models, translational research and clinical investigations, which are not suitable for quantitative aggregation or meta‐analysis. This approach is consistent with the conceptual, mechanistic and rapidly evolving nature of regenerative aesthetics, where scaffold‐based biomaterials, injectable fillers and biological signaling strategies coexist and interact.

### 2.2 Literature Sources

The review prioritized peer‐reviewed experimental, translational, and clinical studies consistently cited across the core literature in regenerative aesthetics, ensuring conceptual coherence and biological relevance. These references comprise narrative and systematic reviews, experimental studies, translational investigations and clinical reports addressing extracellular matrix biology, skin aging, immune‐mediated tissue remodeling, collagen biostimulation, injectable fillers, skin quality therapies and biologically driven regenerative strategies. No external publications beyond those included in the review were introduced, in order to preserve internal consistency and alignment with the foundational literature selected by the authors.

### 2.3 Search Strategy

To describe how the cited literature can be retrieved and organized, a structured search framework was defined reflecting the strategies used across the referenced studies. Searches were conceived for major biomedical databases commonly used in dermatology, plastic surgery, and regenerative medicine, including PubMed/MEDLINE, Scopus, and Web of Science. Search terms were organized around three complementary axes: the biological process of interest, the anatomical context, and the category of intervention.

Descriptors related to regeneration and remodeling included skin regeneration, dermal regeneration, cutaneous regeneration, tissue remodeling and wound healing. These were combined with terms related to extracellular matrix biology, collagen synthesis, mechanotransduction and tissue quality. Intervention‐specific descriptors encompassed injectable fillers and skin quality therapies, such as hyaluronic acid and skinboosters, in addition to particulate collagen biostimulators, including poly‐l‐lactic acid (PLLA), poly‐d,l‐lactic acid (PDLLA), calcium hydroxyapatite (CaHA) and polycaprolactone (PCL), as well as thread‐based scaffolds such as polydioxanone (PDO). Biologically driven regenerative strategies were captured using descriptors such as polynucleotides (PN), polydeoxyribonucleotide (PDRN), exosomes and extracellular vesicles (EVs).

Boolean operators were applied to connect these axes, with syntax adapted to the requirements of each database. Broad queries were used to map the overall regenerative landscape in aesthetic medicine, while more focused strategies targeted specific intervention classes, such as hyaluronic acid–based skinboosters, scaffold‐induced neocollagenesis, mechanotransduction mediated by absorbable threads, and signaling‐based bioregenerative therapies. The descriptors used to structure database searches are summarized in Table 1, while representative Boolean search strategies are presented in Table 2.

**TABLE 1:** Search keywords/descriptors used to structure database queries.

Search domain	Keywords/descriptors
Regeneration core	Skin regeneration; dermal regeneration; cutaneous regeneration; tissue regeneration; wound healing; tissue remodeling; regenerative aesthetics
ECM and remodeling	Extracellular matrix; ECM remodeling; collagen remodeling; elastin; proteoglycans; matrix organization; matrix metalloproteinases; neocollagenesis
Aging context	Skin aging; intrinsic aging; extrinsic aging; photoaging; oxidative stress; fibroblast senescence
Injectable fillers/skin quality	Hyaluronic acid; HA filler; skinboosters; intradermal hyaluronic acid; skin quality improvement
Collagen biostimulation	Collagen biostimulator; collagen induction; collagen inducer; biostimulatory filler; foreign body response; subclinical inflammation
PLLA	Poly‐l‐lactic acid; PLLA; poly‐l‐lactide; injectable PLLA
PDLLA	Poly‐d,l‐lactic acid; PDLLA; poly(d,l‐lactide); injectable PDLLA
CaHA	Calcium hydroxyapatite; CaHA; hydroxyapatite microspheres
PCL	Polycaprolactone; PCL; PCL microspheres; collagen stimulator
PDO	Polydioxanone; PDO; PDO threads; absorbable threads; thread lifting
Bioregenerators	Polynucleotides; polydeoxyribonucleotide; PDRN; purinergic signaling
Exosomes/EVs	Exosomes; extracellular vesicles; small extracellular vesicles; cell‐free therapy

*Source:* Elaborated by the author.

**TABLE 2:** Representative Boolean search strategies used in the narrative review.

Search focus	Boolean search string
Generic regeneration and interventions (broad)	(regeneration OR regenerative OR remodeling) AND (skin OR dermal OR cutaneous) AND (biostimulator OR biomaterial OR filler OR thread OR bioregenerator)
Injectable fillers and skin quality therapies	(“hyaluronic acid” OR skinbooster* OR HA) AND (skin OR dermal OR facial) AND (regeneration OR remodeling OR collagen OR elasticity)
Particulate collagen biostimulators (focused)	(PLLA OR PDLLA OR “poly‐l‐lactic acid” OR “poly‐d,l‐lactic acid” OR CaHA OR “calcium hydroxyapatite” OR PCL OR polycaprolactone) AND (skin OR dermal OR facial) AND (collagen OR neocollagenesis OR remodeling)
Thread‐based biomaterials (PDO)	(PDO OR polydioxanone OR “absorbable thread” OR “thread lift”) AND (dermal OR skin) AND (collagen OR remodeling OR angiogenesis)
Bioregenerators and biological signaling	(polynucleotide* OR PDRN OR “polydeoxyribonucleotide” OR exosome* OR “extracellular vesicle*”) AND (skin OR dermal) AND (regeneration OR repair OR remodeling OR angiogenesis)

*Source:* Elaborated by the author.

### 2.4 Eligibility Criteria

Eligibility for inclusion in this narrative synthesis followed qualitative selection principles rather than formal systematic screening. Publications were considered relevant when they provided mechanistic, histological, translational, or clinically interpretable information on cutaneous regeneration, extracellular matrix remodeling, immune modulation associated with injectable fillers or biomaterials, or clinical outcomes related to collagen biostimulation, skin quality improvement, and bioregenerative strategies. Studies were excluded if they lacked translational relevance to skin biology, focused exclusively on surgical technique without biological or regenerative analysis, addressed non‐cutaneous tissues, or were not included in the review's reference list.

### 2.5 Data Synthesis

Data handling was qualitative and interpretative. Instead of extracting numerical endpoints, the cited literature was analyzed through thematic synthesis emphasizing biological mechanisms of action, interactions between injectable fillers or biomaterials and host tissue, immune and extracellular matrix modulation, integration of dermal and hypodermal compartments, and clinical implications such as durability, tissue quality, elasticity, and biomechanical behavior. Evidence was conceptually grouped into domains encompassing extracellular matrix biology, aging‐related impairment of regenerative capacity, hyaluronic acid–based skin quality therapies, scaffold‐based collagen biostimulation, immune resolution and macrophage phenotype, dermal–adipose interactions, and biological bioregenerators.

### 2.6 Critical Appraisal

Critical appraisal was performed using an interpretative framework appropriate for narrative reviews. Formal risk‐of‐bias assessment tools were not applied. Instead, emphasis was placed on study design, biological plausibility, translational relevance, internal coherence and reproducibility across experimental and clinical contexts. Particular attention was given to distinguishing well‐established mechanisms, such as hyaluronic acid–mediated mechanotransduction and controlled inflammatory neocollagenesis induced by particulate biomaterials, from emerging and less standardized approaches involving biological signaling agents and extracellular vesicle–based therapies.

### 2.7 Scope and Methodological Limitations

This methodology prioritizes conceptual depth, coherence, and alignment with foundational literature in regenerative aesthetics over exhaustive coverage. Although this approach may exclude more recent publications not present in the source material, it supports a focused and biologically grounded narrative consistent with the objectives of a narrative review in aesthetic and reconstructive medicine.

## 3 Results

### 3.1 Regeneration as a Multilevel Cutaneous Process

The literature analyzed consistently characterizes cutaneous regeneration as a complex and multilevel biological process involving extracellular matrix (ECM) remodeling, immune regulation, angiogenesis and coordinated interactions between dermal and hypodermal compartments. Narrative and experimental studies converge in describing regeneration as an active, biologically instructed phenomenon rather than a passive consequence of tissue injury or aesthetic intervention [8, 10, 11]. Central to this process is the ECM, which functions not only as a structural scaffold but also as a dynamic signaling platform capable of regulating fibroblast behavior, immune cell recruitment and vascular responses [4, 5, 6]. The main biological mechanisms involved in cutaneous regeneration, as described in the reviewed literature, are summarized in Table 3.

**TABLE 3:** Biological mechanisms involved in cutaneous regeneration described in the reviewed literature.

Biological mechanism	Key cellular and molecular events	Main type of evidence	Key references
Extracellular matrix remodeling	Fibroblast activation, collagen I and III synthesis, elastin reorganization, MMP/TIMP balance	Experimental, histological, narrative reviews	[4, 5, 6, 9]
Controlled inflammatory response	Early macrophage recruitment, transition toward pro‐regenerative phenotypes	Experimental, translational	[10, 18]
Angiogenesis	Endothelial activation, VEGF signaling, increased microvascular density	Experimental, clinical	[10, 24]
Mechanotransduction	Conversion of mechanical stress into biochemical signaling	Experimental, histological	[16, 18]
Dermal–hypodermal signaling	Adipocyte–fibroblast cross‐talk, paracrine signaling	Experimental, translational	[7, 16]

*Source:* Elaborated by the author.

Age‐related impairment of regenerative capacity was a recurring finding. Studies addressing intrinsic and extrinsic skin aging consistently reported collagen fragmentation, elastin disorganization, and reduced fibroblast responsiveness, leading to impaired mechanotransduction and diminished capacity for ECM reorganization [8, 9]. These alterations compromise not only dermal biomechanics but also downstream signaling pathways essential for effective tissue remodeling, providing the biological rationale for regenerative interventions targeting matrix restoration rather than simple volume replacement.

### 3.2 Collagen Biostimulators and Scaffold‐Mediated Remodeling

Particulate collagen biostimulators, including poly‐l‐lactic acid (PLLA), poly‐d,l‐lactic acid (PDLLA), calcium hydroxyapatite (CaHA) and polycaprolactone (PCL), were consistently associated with induction of neocollagenesis and progressive ECM remodeling through a controlled inflammatory cascade. Experimental, histological and clinical studies demonstrated that these materials act as temporary scaffolds, initiating an innate immune response characterized by macrophage recruitment, followed by fibroblast activation and deposition of newly synthesized collagen, predominantly types I and III [12, 18, 19].

PLLA was the most extensively investigated material in the reviewed literature. Both experimental and clinical studies reported gradual polymer degradation accompanied by sustained collagen deposition, dermal thickening and improvement in tissue quality, with clinical outcomes becoming more evident over time rather than immediately after injection [12, 20]. PDLLA demonstrated similar regenerative behavior, with differences attributed to polymer crystallinity and degradation kinetics influencing the temporal profile of collagen induction [21].

CaHA‐based injectables were described as inducing a robust but controlled inflammatory response around calcium microspheres, leading to organized collagen deposition and dermal reinforcement without excessive foreign‐body reaction [19]. PCL fillers were associated with prolonged ECM remodeling and long‐lasting regenerative effects, attributed to slower degradation and sustained mechanical stimulation of surrounding tissues [21]. A comparative overview of particulate biostimulators, their mechanisms, types of evidence and clinical applications is presented in Table 4.

**TABLE 4:** Regenerative interventions in aesthetic medicine: Materials, mechanisms, evidence and clinical applications.

Intervention/Material	Category	Main mechanisms of action	Type of studies	Key regenerative findings	Key references
Poly‐l‐lactic acid (PLLA)	Particulate collagen biostimulator	Scaffold‐induced controlled inflammation; macrophage recruitment; fibroblast activation	Experimental, histological, clinical	Progressive neocollagenesis; dermal thickening; ECM reorganization	[12, 18, 20]
Poly‐d,l‐lactic acid (PDLLA)	Particulate collagen biostimulator	Similar to PLLA with distinct degradation kinetics	Experimental, clinical	Sustained collagen induction; long‐term remodeling	[21]
Calcium hydroxyapatite (CaHA)	Particulate collagen biostimulator	Microsphere‐induced fibroblast activation; angiogenic support	Histological, clinical	Organized collagen deposition; dermal reinforcement	[18, 19]
Polycaprolactone (PCL)	Long‐lasting collagen biostimulator	Prolonged scaffold presence; sustained mechanical stimulation	Experimental, clinical	Durable collagen remodeling	[21]
Hyaluronic acid fillers	Injectable biomechanical modulator	Hydration; viscoelastic support; mechanotransduction	Clinical, narrative reviews	Improved elasticity and ECM function	[8, 9]
Skinboosters (intradermal HA)	Skin quality therapy	ECM hydration; fibroblast stimulation via mechanical cues	Clinical, experimental	Improved texture, luminosity and elasticity	[8]
Polydioxanone (PDO) threads	Thread‐based scaffold	Mechanical tension; mechanotransduction; localized inflammation	Experimental, histological, clinical	Increased collagen density; angiogenesis	[18]
Polynucleotides (PN)	Biological bioregenerator	Purinergic signaling; fibroblast migration	Experimental, clinical	ECM modulation; angiogenesis	[24]
Polydeoxyribonucleotide (PDRN)	Biological bioregenerator	Adenosine A2A receptor activation; anti‐inflammatory signaling	Experimental, clinical	Tissue repair; inflammation resolution	[24]
Exosomes/extracellular vesicles	Cell‐free bioregenerator	Transfer of proteins, lipids and miRNA	Experimental	Dermal regeneration; photoaging improvement	[23]

*Source:* Elaborated by the author.

### 3.3 Hyaluronic Acid, Skinboosters and Microenvironmental Modulation

Hyaluronic acid (HA)—based injectables and skinboosters were consistently described as modulators of the dermal microenvironment rather than primary drivers of neocollagenesis. Clinical and narrative studies reported improvements in skin hydration, elasticity, and surface texture following intradermal or superficial HA administration, reflecting enhanced ECM hydration and viscoelastic properties [8].

Mechanistic evidence indicated that HA influences fibroblast behavior through mechanotransduction and receptor‐mediated pathways, while its degradation products may further stimulate matrix turnover [9]. When used as skinboosters, HA was associated predominantly with improvements in skin quality parameters rather than volumetric correction, positioning these interventions as supportive components within the regenerative continuum. These findings and their clinical implications are summarized alongside other regenerative interventions in Table 4.

### 3.4 Thread‐Based Biomaterials and Mechanotransduction

Polydioxanone (PDO) threads were consistently reported to exert regenerative effects through the combined action of mechanical tension and biologically mediated tissue remodeling. Experimental, histological, and clinical studies demonstrated increased collagen density, fibroblast activation, and angiogenesis along the trajectory of implanted threads, with these effects persisting beyond polymer resorption [18].

Mechanotransduction emerged as a central mechanism, whereby mechanical forces generated by thread placement are converted into biochemical signals that promote ECM remodeling and vascular responses. These observations support the use of PDO threads not only for mechanical lifting but also as biologically active tools for tissue regeneration. The regenerative role of PDO threads is detailed in Table 4.

### 3.5 Bioregenerative Agents and Signaling‐Based Regeneration

Bioregenerative agents, including polynucleotides, polydeoxyribonucleotide (PDRN) and extracellular vesicles, were described as acting predominantly through biological signaling rather than scaffold formation. Experimental and clinical studies reported enhanced angiogenesis, fibroblast migration, and modulation of inflammatory responses following PN and PDRN administration, frequently linked to purinergic signaling and activation of adenosine A2A receptors [24].

Extracellular vesicles and exosomes were characterized as emerging cell‐free regenerative platforms capable of modulating inflammation, oxidative stress and intercellular communication through transfer of proteins, lipids and microRNAs [23]. Although experimental models demonstrated improvements in dermal regeneration and photoaging parameters, the literature highlighted substantial heterogeneity in vesicle sources, isolation methods and dosing strategies. A synthesis of biological bioregenerators, mechanisms and current evidence is included in Table 4, while their translational maturity is addressed in Table 5.

**TABLE 5:** Level of evidence, biological robustness and translational maturity of regenerative strategies in aesthetic medicine.

Regenerative strategy	Predominant level of evidence	Consistency of biological mechanism	Translational maturity	Predictability of clinical outcomes	Standardization of protocols
Particulate collagen biostimulators (PLLA, PDLLA, CaHA, PCL)	Experimental, histological, prospective clinical studies	High—well‐characterized inflammatory cascade and fibroblast activation	Established	High when proper technique and indication are respected	Moderate to high
Polydioxanone (PDO) threads	Experimental, histological, clinical observational studies	Moderate to high—mechanotransduction and localized inflammation	Established	Moderate to high	Moderate
Hyaluronic acid fillers	Clinical trials, observational studies, narrative reviews	High—hydration, viscoelasticity and mechanotransduction	Established	High	High
Skinboosters (intradermal HA)	Clinical studies, experimental models	Moderate—microenvironmental modulation	Established	High for skin quality parameters	High
Polynucleotides/PDRN	Experimental and clinical studies	Moderate—purinergic signaling and angiogenesis	Expanding	Moderate	Low to moderate
Exosomes/extracellular vesicles	Experimental and preclinical studies	Variable—dependent on source and isolation method	Emerging	Variable	Low

*Source:* Elaborated by the author.

### 3.6 Integration of Dermal and Hypodermal Compartments

Several studies emphasized the active role of the hypodermis in skin regeneration. Experimental and translational evidence demonstrated that adipose tissue contributes to regenerative processes through paracrine signaling, immune modulation, and support of fibroblast function [7, 16]. Interventions capable of influencing both dermal ECM remodeling and hypodermal biology were associated with more sustained improvements in tissue quality, elasticity, and biomechanical behavior. The broader clinical implications of these integrated regenerative mechanisms are summarized in Table 6.

**TABLE 6:** Clinical implications and practical considerations derived from regenerative interventions.

Clinical domain	Key findings from the reviewed literature	Practical implications for clinical practice
Concept of regeneration	Regeneration involves ECM remodeling, immune modulation and vascular support	Treatments should aim at biological restoration rather than volumetric correction alone
Tissue quality improvement	Skin quality outcomes correlate with matrix organization and hydration	Combination of biostimulators and skin quality therapies may enhance results
Longevity of outcomes	Duration of effect depends on scaffold persistence and remodeling kinetics	Product selection should consider degradation profile and desired longevity
Safety profile	Controlled inflammation is essential for regeneration	Overcorrection and excessive inflammatory response must be avoided
Combination therapies	Different regenerative mechanisms are complementary	Sequential or combined protocols may produce synergistic effects
Patient selection	Aging degree and tissue quality influence outcomes	Individualized treatment planning is essential
Future clinical application	Biological signaling strategies are expanding	Need for standardized protocols and long‐term outcome assessment

*Source:* Elaborated by the author.

## 4 Discussion

### 4.1 Regeneration in Aesthetic Medicine: From Aspirational Rhetoric to Biological Accountability

The progressive adoption of the term *regeneration* in aesthetic medicine reflects a profound transformation of the field, which has moved from a predominantly corrective and volumetric approach toward biologically oriented strategies aimed at restoring tissue quality, function, and long‐term adaptability. This shift is legitimate and is supported by advances in biomaterials science, extracellular matrix (ECM) biology, tissue immunology, and mechanobiology. Nevertheless, the scientific literature consistently shows that the expansion of the term *regenerative* has occurred faster than the conceptual maturation required to sustain it, leading to semantic overlap among regeneration, repair, remodeling, and biostimulation [4, 5, 10].

From a classical biological standpoint, regeneration implies complete restitution of tissue architecture, cellular composition, and function in a manner indistinguishable from native pre‐injury tissue. In adult organisms—particularly in complex tissues such as human skin—this form of morphogenetic regeneration is exceptional. The predominant process following injury or intervention is repair, characterized by ECM remodeling, collagen reorganization, biomechanical changes, and partial functional recovery [6, 9]. Overlooking this biological constraint has contributed to the trivialization of the regeneration concept, which is often equated with mere induction of neocollagenesis or increased dermal thickness.

In this context, the conceptual contribution of Tornnad et al. [25] represents a relevant milestone by proposing a redefinition of regeneration in aesthetic medicine based on mechanisms and function rather than exclusively on morphological criteria. Under this framework, regeneration should be understood as the sustained restoration of tissue function, ECM organization, biomechanical behavior, and biological signaling through activation of endogenous regenerative pathways, even when complete histologic restitution is not achieved. This definition shifts the evaluative focus from immediate visual aesthetics to tissue biology, introducing a standard of scientific accountability that is essential for the field's evolution [25].

### 4.2 The Extracellular Matrix as the Central Axis of Cutaneous Regeneration

Among the elements discussed across the literature, the ECM emerges as the primary regulator of cutaneous regeneration. Far from being a passive structural scaffold, the ECM constitutes a dynamic signaling platform governing cell adhesion, migration, differentiation, angiogenesis, immune modulation, and mechanotransduction [4, 5]. In skin, fibroblast phenotype and activity are strongly modulated by matrix composition, organization, and stiffness; consequently, structural ECM alterations directly affect the tissue's regenerative capacity.

Cutaneous aging—both intrinsic and extrinsic—is characterized by collagen fragmentation, elastic fiber disorganization, reduction in glycosaminoglycans, and disruption of the matrix's three‐dimensional architecture. These changes impair mechanotransduction, resulting in loss of mechanical cues required to maintain fibroblast activity, reduced collagen synthesis, and increased expression of matrix metalloproteinases [8, 9]. In this scenario, interventions capable of partially restoring ECM organization and biomechanical integrity may reactivate regenerative circuits even in the absence of full morphogenetic regeneration.

### 4.3 Tissue Immunology: The Balance Between Regeneration and Fibrosis

Another critical axis of regeneration is the immune response. Tissue regeneration does not occur in the absence of inflammation; rather, it depends on an initially controlled inflammatory phase followed by efficient resolution. Macrophages play a central role in this process and may adopt pro‐regenerative or pro‐fibrotic phenotypes depending on the stimulus, microenvironment, and duration of exposure [10].

Injectable biomaterials inevitably interact with the innate immune system, eliciting foreign‐body responses of variable intensity. The quality of this response is decisive for biological outcomes. Excessive or persistent inflammation favors fibrous encapsulation and functional loss, whereas a modulated response supports constructive remodeling and ECM reorganization [14, 26]. Therefore, the mere presence of neocollagenesis cannot be interpreted as regeneration if it is accompanied by a disorganized or chronic inflammatory profile.

### 4.4 Mechanotransduction and the Dermal–Hypodermal Unit

Mechanotransduction integrates ECM biology, immunology, and cellular behavior. Mechanical forces generated by tissue stretching, volumetric filling, or thread traction are converted into biochemical signals that regulate cellular proliferation, angiogenesis, and matrix remodeling [9]. This principle explains why interventions traditionally considered structural may elicit durable biological effects.

Moreover, growing evidence indicates that cutaneous regeneration cannot be understood in isolation at the dermal level. Subcutaneous adipose tissue acts as an endocrine and immunologic organ, producing adipokines, cytokines, and angiogenic factors that directly influence dermal behavior [7, 27]. Interactions between dermis and hypodermis—including sWAT and dWAT—appear to be determinants for the durability and quality of regenerative outcomes, although this dimension remains underexplored in clinical literature.

### 4.5 Particulate Biostimulators and PDO Threads: Mechanistic Evidence, “Regenerative” Findings, and the Role of Subcutaneous Tissue

Particulate biostimulators—particularly PLLA, PDLLA, CaHA, and PCL—represent the most thoroughly characterized branch of so‐called “regenerative” aesthetics because, unlike purely volumetric fillers, they function as temporary bioactive scaffolds capable of triggering a relatively predictable sequence of events: regulated innate immune response, recruitment and organization of macrophages and foreign‐body giant cells, fibroblast activation, matrix deposition, and progressive remodeling [12, 14, 18, 28]. Still, what is often described as “regeneration” in practice corresponds, in most cases, to functional reparative remodeling—namely, sustained improvement in structural and biomechanical parameters without complete restitution of native cutaneous architecture, particularly adnexal structures and original fibrillar patterns [25].

A crucial—and sometimes underestimated—point is that tissue responses to particulates are not exclusively dermal. Skin functions as an integrated dermal–hypodermal unit, and increasingly robust evidence indicates that the hypodermis, particularly subcutaneous adipose tissue (including functional compartments such as dWAT/sWAT), actively participates in homeostasis, repair, and “skin quality” through endocrine, paracrine, and immunometabolic mechanisms [7, 27, 29]. dWAT, for example, is now understood not merely as a mechanical reservoir but as a dynamic compartment that influences inflammation, follicular regeneration, antimicrobial defense, and dermal niche behavior through lipid signals and cytokines—phenomena that help explain why interventions altering matrix and microenvironment may also affect subcutaneous thickness and behavior [7, 27].

Within this framework, comparisons among particulate biostimulators should consider three dimensions: (i) the nature of the stimulus (chemical/biocue, mechanical, and inflammatory), (ii) the temporal profile of degradation and tissue response, and (iii) the quality/architecture of the newly formed tissue and its integration with the subcutaneous compartment [12, 18, 25].

PLLA remains the biostimulator with the most consistent body of clinical and histologic evidence in humans for progressive neocollagenesis, thickening, and remodeling within a prolonged response window, consistent with its degradation profile and the cumulative character of treatment sessions [12, 30]. Beyond “collagen quantity,” recent work emphasizes that the critical determinant is collagen quality and organization—whether the tissue progresses toward constructive remodeling with improved biomechanics or trends toward a more disorganized and less functional pattern [12, 18]. Additional mechanistic evidence suggests that polylactides may modulate pathways related to oxidative stress and matrix preservation (e.g., involving antioxidant axes), which is biologically plausible in aged tissues where oxidative stress and collagen fragmentation perpetuate fibroblast dysfunction [9, 31, 32].

PDLLA shares the scaffold principle and controlled inflammatory stimulation but presents physicochemical particularities that affect degradation kinetics, immune cell interactions, and potentially the pattern of matrix deposition. Contemporary interest in PDLLA derives partly from the attempt to obtain biological responses with specific temporal profiles and favorable tissue integration; however, compared with PLLA, there remains a lower density of standardized histologic data and fewer longitudinal studies with objective endpoints comparable across populations and techniques [33, 34]. This creates a scenario in which biological plausibility is high, but evidentiary weight still depends on methodological consolidation.

CaHA occupies a singular position because it combines immediate support with microsphere‐mediated remodeling stimulation, has a relatively broad aesthetic literature, and has been the subject of rigorous conceptual discussion regarding what can (or cannot) be termed regeneration. Mechanistically, CaHA functions as a platform for matrix deposition and local signaling, with evidence of improvement in dermal parameters and, in some studies, measurable tissue thickening and changes compatible with functional improvement [19]. However, the critical contribution of Tonnard et al. [25] is the reminder that neocollagenesis around a particulate material does not automatically equate to restoration of the original tissue: it is reparative remodeling, potentially “regenerative” in a functional sense, but not morphogenetic [25]. The clinical robustness of CaHA is strong for specific indications, yet scientific discourse should remain proportional to demonstrable mechanisms and measured outcomes, avoiding extrapolation beyond what histology and biology support [18, 25].

PCL, in turn, is often discussed as a more persistent scaffold, with prolonged mechanobiological stimulation and potential for more durable remodeling—an hypothesis coherent with its degradation profile. Recent literature has examined both its impact on “skin quality” and its interactions with deeper compartments, suggesting that part of its clinical effect may involve microenvironmental reorganization and structural support with repercussions at the dermal–hypodermal interface [35, 36]. Still, when attempting a direct comparison of “which is more regenerative,” PCL faces a typical limitation of emerging technologies: there is growing interest and promising data, yet large standardized comparative datasets and robust, replicable histofunctional endpoints remain lacking [35, 37].

When the question is framed as “what is most regenerative among the particulates,” the answer depends on how regeneration is defined and which evidentiary dimensions are prioritized. If one adopts the perspective of functional regeneration—sustained improvement in biomechanical behavior, thickness, and matrix quality with tissue integration—PLLA tends to offer an advantage by combining long clinical experience, stronger histologic/longitudinal support, and temporal coherence of remodeling [12, 30]. CaHA has a sound rationale and a solid evidence base for remodeling with functional gains, but requires terminological precision and caution in claims of “full” regeneration, as underscored by Tonnard et al. [25]. PCL has substantial potential for prolonged remodeling and emerging findings suggest meaningful effects, including tissue thickness and structural quality, but comparative maturity remains limited. PDLLA, while biologically plausible and increasingly used clinically, requires greater density of standardized and comparative studies to assume a higher position within this hierarchy with confidence. In summary, if “more regenerative” is interpreted as stronger evidentiary support for durable functional remodeling, PLLA often stands out; if interpreted as capacity to modulate microenvironment with specific cues, CaHA and PCL become competitive, but the strength of conclusions remains constrained by methodological heterogeneity and divergent endpoints (Table 7) [12, 18, 25].

**TABLE 7:** Mapping of mechanisms of action, evidence gaps, and involvement of dWAT/sWAT across biomaterials used in regenerative aesthetics.

Biomaterial	Primary mechanism of action	Main biological targets	Involvement of dWAT/sWAT	Level and nature of evidence	Key evidence gaps	Regenerative interpretation
PLLA (Poly‐l‐lactic acid)	Bioactive particulate scaffold inducing controlled foreign‐body response, macrophage recruitment, fibroblast activation, and progressive neocollagenesis	Dermal fibroblasts, ECM remodeling pathways, macrophage‐mediated signaling	Indirect but plausible activation of dermal–hypodermal crosstalk; potential modulation of adipose‐derived paracrine signals and structural support of dWAT	Robust clinical, histological, and longitudinal human studies	Limited direct evaluation of adipose remodeling; scarce studies specifically addressing dWAT/sWAT responses	Strongest evidence for functional regenerative remodeling involving dermal dominance with probable hypodermal contribution
PDLLA (Poly‐d,l‐lactic acid)	Particulate scaffold with controlled inflammatory stimulation and fibroblast activation; distinct degradation kinetics	Fibroblasts, innate immune cells, ECM deposition	Hypothesized indirect interaction with hypodermal immune–metabolic signaling; poorly characterized	Moderate clinical evidence; limited histological data	Lack of direct studies assessing adipose tissue response; insufficient mechanistic linkage to dWAT/sWAT	Potential regenerative remodeling, hypodermal involvement remains speculative
CaHA (Calcium hydroxyapatite)	Microsphere‐based scaffold inducing collagen deposition around particles; immediate mechanical support plus delayed fibroplasia	Fibroblasts, ECM deposition around microspheres; mechanotransduction	Minimal direct evidence of adipose remodeling; possible mechanical influence at dermal–hypodermal interface	Extensive clinical use; good imaging and clinical outcomes	Absence of data on adipocyte biology, dWAT signaling, or adipose ECM integration	Reparative functional remodeling, primarily dermal
PCL (Polycaprolactone)	Long‐lasting biodegradable scaffold providing prolonged mechanobiological stimulation	Fibroblasts, ECM, prolonged mechanotransduction pathways	Emerging evidence suggests potential modulation of subcutaneous support and dermal–hypodermal interface due to prolonged scaffold persistence	Growing clinical interest; limited mechanistic studies	Insufficient direct assessment of adipose compartment changes; lack of adipose‐focused endpoints	Promising regenerative remodeling, including possible hypodermal contribution
Hyaluronic acid (HA)/Skinboosters	Hydration‐based ECM modulation; indirect mechanotransduction	Dermal ECM hydration, fibroblast mechanosensing	No direct regenerative interaction; transient volumetric or hydration‐related effects may minimally influence sWAT mechanics	Very strong clinical evidence for skin quality	No evidence of adipose signaling modulation or structural hypodermal effects	Microenvironment modulator, not regenerative
PDO threads	Local mechanotransduction via tensile forces and controlled inflammation along thread trajectory	Fibroblasts, local angiogenesis	Possible localized mechanical influence on superficial hypodermis depending on depth; not systematically studied	Moderate clinical evidence; observational studies	High technique dependence; no standardized assessment of adipose response	Localized mechanobiological remodeling, limited regenerative depth
Polynucleotides/PDRN	Activation of adenosine A2A receptors; angiogenesis, fibroblast migration, immune modulation	Endothelial cells, fibroblasts, immune cells	Biologically plausible modulation of adipose immune environment and vascular niche; no direct aesthetic studies	Strong experimental data; limited aesthetic clinical evidence	Absence of human data assessing adipose tissue or dermal–hypodermal integration	Biological regenerative signaling, underexplored in adipose context
Extracellular vesicles/Exosomes	Direct cell‐to‐cell signaling via proteins, lipids, miRNAs	Fibroblasts, endothelial cells, immune cells, adipose‐derived stromal cells	High theoretical relevance for dWAT/sWAT regulation, adipose–dermal signaling, and immune resolution	Strong preclinical and mechanistic evidence	Lack of standardization and human translational data targeting adipose compartments	Closest to true regeneration conceptually, currently experimental

This comparison must be expanded by incorporating the subcutaneous compartment as an active component of the regenerative phenomenon. Work in cutaneous and adipose biology supports that adipocytes, stromal cells, and adipose tissue architecture directly influence vascularization, inflammation, and dermal behavior, with implications for “skin quality” and durability of outcomes [7, 27]. Recent literature also points to mechanisms through which interventions may increase thickness and support not only of the dermis but also of subcutaneous compartments, which are clinically relevant in laxity and structural aging [38, 39]. In this sense, approaches that promote constructive remodeling with better dermal–hypodermal integration are more consistent with functional regeneration than those that merely generate temporary volume or superficial effects.

Overall, the literature suggests that particulate biomaterial–based regenerative aesthetics is most coherent when it explicitly acknowledges that its effects largely reflect functional reparative remodeling—and that “regenerativity” depends less on labeling and more on objective demonstration of biomechanical improvement, matrix integration, and, ideally, impact on the dermal–hypodermal unit with controlled inflammation and effective resolution [7, 9, 18, 25, 27].

### 4.6 Hyaluronic Acid and Skinboosters: High Clinical Robustness, Limited Regenerative Depth

Hyaluronic acid (HA) is unequivocally the most extensively studied injectable biomaterial in contemporary aesthetic medicine, with a high level of clinical evidence supporting improvements in hydration, elasticity, viscoelasticity, and skin texture. Clinical trials, observational studies, and narrative reviews consistently show measurable benefits in skin quality, particularly when HA is administered intradermally in low‐density formulations or as skin boosters [8, 40, 41, 42].

Biologically, HA exerts its effects predominantly through modulation of the dermal microenvironment. Its high hygroscopic capacity contributes to restoration of interstitial water content, improved nutrient diffusion, and optimization of tissue biomechanical behavior. In addition, HA interactions with cellular receptors such as CD44 and RHAMM indirectly influence fibroblast activity, cellular migration, and transient reorganization of the extracellular matrix [9, 43].

Experimental studies indicate that HA may modulate mechanotransduction by restoring local mechanical tension, reactivating intracellular pathways related to collagen synthesis in senescent fibroblasts. However, this stimulus is fundamentally indirect and dependent on the physical presence of the material, and is not associated with sustained activation of endogenous regenerative pathways or deep structural reorganization of the ECM [8, 9].

Accordingly, although there are reports of modest secondary collagen induction with HA—particularly related to needle microtrauma and mechanical microenvironment changes—the literature does not support HA as a driver of tissue regeneration in either a functional or structural sense. Its effects are transient, directly linked to enzymatic polymer degradation and the progressive loss of biomechanical support. There is no consistent evidence that isolated HA use restores native dermal architecture, promotes sustained fibrillar reorganization, or induces immunobiological reprogramming [41, 42]. Therefore, under a conceptually rigorous perspective, HA should be classified as a biological modulator of the cutaneous microenvironment rather than a regenerative agent. Its most relevant clinical role lies in optimizing the “biological terrain,” preparing tissue to respond more efficiently to interventions with greater potential for functional remodeling, such as particulate biostimulators or signaling‐based therapies.

### 4.7 PDO Threads: Mechanobiological Remodeling Dependent on Technique and Tissue Context

Polydioxanone (PDO) threads represent a distinct approach within regenerative aesthetics, grounded predominantly in mechanotransduction. Thread insertion generates local mechanical stimuli associated with a controlled inflammatory response that culminates in fibroblast activation, collagen deposition, and angiogenesis along the thread trajectory [44, 45].

Biologically, studies demonstrate increased collagen density and localized extracellular matrix reorganization in areas adjacent to threads, as well as increased vascularization, suggesting an environment favorable to tissue remodeling. However, unlike injectable biostimulators, the biological stimulus delivered by threads is highly localized and strongly dependent on technical factors such as insertion depth, traction vector, number of threads, applied tension, and pre‐existing tissue biomechanical characteristics [45, 46].

This technical dependence introduces considerable variability in clinical outcomes and limits reproducibility across studies. The available literature is composed predominantly of case series, observational studies, and short‐term assessments, with a scarcity of controlled trials and standardized histologic analyses. Furthermore, reported endpoints often prioritize lifting or support effects rather than deeper functional or regenerative metrics.

From a biological standpoint, PDO threads promote localized mechanobiological remodeling with functional impact restricted to the treated area. There is no compelling evidence of systemic activation of regenerative pathways, global ECM reorganization, or consistent integration with the biology of the dermal–hypodermal unit. Thus, while PDO threads may contribute to focal functional improvement and local tissue quality, they should not be interpreted as comprehensive regenerative agents but rather as adjunctive tools within combined strategies.

### 4.8 Biological Bioregenerators: Mechanistic Coherence Exceeding Available Clinical Evidence

Biological bioregenerators—including polynucleotides, polydeoxyribonucleotide (PDRN), and extracellular vesicles—represent the approach most conceptually aligned with regeneration in a biological sense, as they act directly on cellular signaling pathways, immune modulation, and tissue repair without primary reliance on scaffolds or mechanical stimuli.

PDRN has been extensively investigated in tissue repair models, demonstrating activity via adenosine A2A receptor activation, with subsequent promotion of angiogenesis, fibroblast migration, increased collagen synthesis, and modulation of inflammatory responses [24, 47]. These mechanisms are biologically coherent with regenerative processes, particularly regarding inflammatory resolution and restoration of tissue homeostasis.

Nevertheless, despite this mechanistic coherence, clinical evidence in aesthetics remains limited. Available studies often involve small samples, heterogeneous formulations, variable protocols, and predominantly subjective or short‐term endpoints. Therefore, although early results are promising, the literature does not yet allow definitive conclusions regarding the magnitude, durability, or predictability of regenerative effects in humans.

Extracellular vesicles, particularly exosomes, represent the closest concept to true regeneration discussed in the current literature. By transferring proteins, lipids, and microRNAs, these structures can directly modulate cellular, epigenetic, and immunological pathways involved in tissue regeneration [23, 48]. Experimental studies demonstrate relevant effects on angiogenesis, reduction of oxidative stress, and modulation of inflammation.

However, clinical translation faces substantial obstacles. The absence of standardization regarding cellular source, isolation methods, molecular characterization, dose, and routes of administration generates profound heterogeneity across studies, compromising reproducibility, safety, and comparability. Thus, despite significant biological promise, extracellular vesicle–based approaches should currently be regarded as experimental or translational tools rather than clinically consolidated regenerative therapies.

### 4.9 Literature Gaps, Methodological Limitations, and Future Directions

Critical appraisal of the literature reveals major structural gaps. Inconsistent definitions of regeneration persist, functional standardized endpoints remain scarce, there are few direct comparative studies among biomaterials, and the biology of the dermal–hypodermal unit has been insufficiently incorporated into investigative models [10, 18, 27]. Many studies prioritize immediate aesthetic endpoints, neglecting parameters such as ECM organization, biomechanical behavior, vascularization, and immune resolution (Table 7).

Advancement of regenerative aesthetics requires more sophisticated metrics capable of capturing functional and biological restoration rather than only visual improvement. Longitudinal studies, standardized histologic analyses, and tighter integration between basic biology and clinical practice will be decisive for consolidating the field.

### 4.10 Clinical Implications and Responsible Use of the Term “Regenerative”

From a clinical standpoint, particulate biostimulators currently represent the most defensible application when described as agents of sustained functional remodeling. Hyaluronic acid remains indispensable as a microenvironmental modulator, PDO threads as adjunctive mechanobiological tools, and biological bioregenerators as promising strategies that still require rigorous validation.

The consolidation of regenerative aesthetics will depend less on indiscriminate expansion of terminology and more on alignment between biology, scientific evidence, and ethical communication. Only in this way can the concept of regeneration evolve from aspirational rhetoric to a scientifically grounded clinical practice.

## 5 Conclusion

This review highlights that regeneration in aesthetic medicine should be understood as a functional, biologically driven process, rather than as a simple consequence of volumetric correction or isolated neocollagenesis. Current evidence supports a paradigm shift toward interventions that modulate the extracellular matrix, mechanotransduction, immune balance, and dermal–hypodermal interactions to promote sustained improvements in tissue quality and function. Within this framework, particulate biostimulators represent the most biologically substantiated tools for long‐term functional remodeling, particularly when their effects are described with conceptual precision rather than overstated regenerative claims. In contrast, hyaluronic acid–based products primarily act as microenvironmental modulators, PDO threads provide localized mechanobiological remodeling, and biological bioregenerators—although mechanistically compelling—still require rigorous clinical validation.

Overall, the literature underscores the importance of biological accountability and conceptual precision in regenerative aesthetics. Regeneration should be framed by mechanism, durability, and functional integration—especially across the dermal–hypodermal unit—rather than by transient morphological outcomes. Clinically, the most responsible approach lies in combining modalities based on their true biological roles, aligning therapeutic strategies with tissue physiology and evidence rather than marketing narratives. The future of regenerative aesthetics will depend on deeper integration between basic biology and clinical practice, ensuring that regenerative claims are scientifically grounded, ethically communicated, and clinically meaningful.

## Funding

The author has nothing to report.

## Conflicts of Interest

A.d.P.B. is affiliated as speaker for Galderma Aesthetics Brazil. The author declares no conflicts of interest regarding the content of this manuscript.

## Data Availability

The data that support the findings of this study are available from the corresponding author upon reasonable request.
